# Variability in the Method of Gastrostomy Placement in Children

**DOI:** 10.3390/children7060053

**Published:** 2020-06-01

**Authors:** Jose H. Salazar, Charles Spanbauer, Manu R. Sood, John C. Densmore, Kyle J. Van Arendonk

**Affiliations:** 1 Division of Pediatric Surgery, Department of Surgery, Medical College of Wisconsin, Children’s Wisconsin, Milwaukee, WI 53226, USA; jsalazar@chw.org (J.H.S.); jdensmore@chw.org (J.C.D.); 2 Division of Biostatistics, Medical College of Wisconsin, Milwaukee, WI 53226, USA; cspanbauer@mcw.edu; 3 Division of Pediatric Gastroenterology, Department of Pediatrics, Medical College of Wisconsin, Children’s Wisconsin, Milwaukee, WI 53226, USA; msood@mcw.edu

**Keywords:** gastrostomy, children, technique, variability

## Abstract

Although gastrostomy placement is one of the most common procedures performed in children, the optimal technique remains unclear. The purpose of this study was to evaluate variability in the method of gastrostomy tube placement in children in the United States. Patients <18 years old undergoing percutaneous endoscopic gastrostomy (PEG) or surgical gastrostomy (SG) (including open or laparoscopic) from 1997 to 2012 were identified using the Kids’ Inpatient Database. Method of gastrostomy placement was evaluated using a multivariable mixed-effects logistic regression model with a random intercept term and a patient-age random-effect term. A total of 67,811 gastrostomy placements were performed during the study period. PEG was used in 36.6% of entries overall and was generally consistent over time. PEG placement was less commonly performed in infants (adjusted odds ratio [aOR] 0.30, 95%CI 0.26–0.33), children at urban hospitals (aOR: 0.38, 95%CI 0.18–0.82), and children cared for at children’s hospitals (aOR 0.57, 95%CI 0.48–0.69) and was more commonly performed in children with private insurance (aOR 1.17, 95%CI 1.09–1.25). Dramatic variability in PEG use was identified between centers, ranging from 0% to 100%. The random intercept and slope terms significantly improved the model, confirming significant center-level variability and increased variability among patients <1 year old. These findings emphasize the need to further evaluate the safest method of gastrostomy placement in children, in particular among the youngest patients in whom practice varies the most.

## 1. Introduction

Gastrostomy placement is one of the most common procedures performed in children. Despite the frequency of gastrostomy tube placement, the best method of placement is unknown. The most commonly utilized approaches are an open or laparoscopic surgical gastrostomy (SG) and percutaneous endoscopic gastrostomy (PEG).

PEG is now well established as an effective method of gastrostomy placement in children and adults after its initial description by Gauderer in 1980 [1]. While some studies have shown similar safety between PEG and SG in children, several meta-analyses have recently reported a higher rate of complications with PEG [2,3,4]. These safety concerns have been most pronounced among the youngest patients [5,6].

The goals of this study were (1) to measure the use of PEG and SG over time, (2) to identify the patient population in which PEG is most frequently being used, and (3) to identify potential variability in PEG use between centers providing care to children. We hypothesized that (1) the use of PEG in children has increased over time and that (2) the use of PEG varies significantly between centers.

## 2. Methods

### 2.1. Data Source

The Kids’ Inpatient Database (KID) (developed for the Healthcare Cost and Utilization Project [HCUP], Agency for Healthcare Research and Quality) was queried from 1997 to 2012. KID is an all-payer database that is produced every three years and includes a representative sample of all pediatric admissions in the United States. Entries in the database are admissions and not individual patients. The database contains variables to perform weighted calculations and translate results into national estimates [7].

### 2.2. Study Population

All patients <18 years old at admission were included for analysis. Gastrostomy placement was categorized as PEG or SG (including open or laparoscopic) based on procedural International Classification of Diseases-Ninth Revision (ICD-9) codes. PEG was defined as having an ICD-9 code of 43.11 as a primary or secondary procedure. SG was defined as having an ICD-9 code of 43.19 as primary or secondary procedure. ICD-9 codes do not provide technical details about the method of gastrostomy placement other than specifying whether it was with percutaneous endoscopic approach; therefore patients could not be stratified further with regard to clinically relevant technical differences that exist within PEG and SG (e.g., laparoscopic vs. open, push vs. fascial securement). Children’s hospitals were considered any centers defined by the National Association of Children’s Hospitals and Related Institutions as a children’s general hospital, children’s specialty hospital, or children’s unit in a general hospital.

### 2.3. Statistical Analysis

Survey weights were used to generate contingency tables of patient and center characteristics by method of gastrostomy placement. Bivariate associations between these characteristics and use of PEG were compared using t-tests for continuous variables and chi-squared tests of independence for categorical variables. Multivariable mixed-effects logistic regression models were used to test the association of each covariate with the use of PEG, adjusted for the other covariates. To account for both possible heterogeneity in the use of PEG across centers and possible heterogeneity in the effect of patient age across centers, both a random intercept term and a patient-age random-effect term were included in the model. The variable used to identify individual hospitals across multiple years was only available from 2000 to 2009, and thus only those years were included in the regression analyses.

In order to visualize the distribution of gastrostomy placements across centers that performed more than 20 gastrostomies in the years sampled, the proportion of PEG use by center was graphed for all children and for infants. Missing data in the included covariates were handled using the “missing indicator method,” in which missing data are categorized as unknown, thereby allowing patients’ with missing data to still contribute all other data points to the regression analyses [8]. All tests were two-sided, with statistical significance set at α = 0.05. All analyses were performed using STATA 15.1/MP (College Station, Texas).

## 3. Results

### 3.1. Study Population

The analysis included a total of 67,811 gastrostomy tube placements. Overall, SG was more commonly utilized (63.4%) than PEG (36.6%) (Table 1). Infants (<1 year old) accounted for the majority of the study population (52.1%), and PEG placement was considerably less common (27.3%) among infants compared to older children (46.3%). There was a statistically significant difference in the proportion of PEG use over time, but no clear temporal trend was evident. The proportion of PEG use per year was 18.2% in 2000, 19.8% in 2003, 21.0% in 2006, 19.8% in 2009 and 21.1% in 2012 (*p* < 0.001).

Between 2000 and 2009 (calendar years 2000, 2003, 2006, and 2009), there were 816 individual centers that performed gastrostomy placement in children. Of those centers, 246 (30%) performed greater than 20 gastrostomy tube placements total during those years. In total, 492 individual centers performed gastrostomy placement in infants. Of those centers, 188 (38.2%) performed greater than 20 gastrostomy tube placements. In total, 380 individual centers performed PEG placement in infants, and 47 (12.4%) performed greater than 20 PEGs (36 of the 47 were children’s hospitals). Of the centers performing PEG placement in infants, 135 (35.5%) were children’s hospitals.

### 3.2. Characteristics by Gastrostomy Placement Method

Infants (adjusted odds ratio [aOR] 0.30, 95%CI 0.26–0.33), children at urban hospitals (aOR: 0.38, 95%CI 0.18–0.82), and children cared for at children’s hospitals (aOR 0.57, 95%CI 0.48–0.69) were less likely to undergo PEG placement (Table 2). Children with private insurance (aOR 1.17, 95%CI 1.09–1.25) were more likely to undergo PEG placement. Hospital size, teaching status, and geographic region were not significantly associated with the method of gastrostomy placement.

### 3.3. Center-Level Variability in Gastrostomy Placement

**Figure 1:**
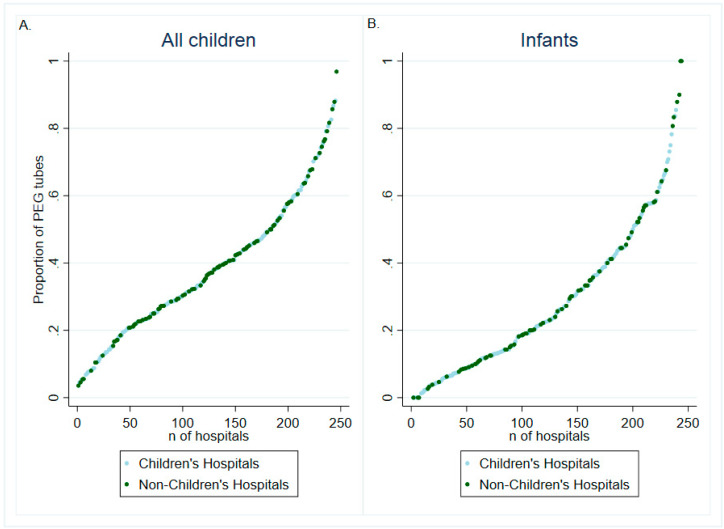
Distribution in the Proportion of Percutaneous Endoscopic Gastrostomy (PEG) Placement across Centers Performing >20 Gastrostomy Tube Placements in (**A**) Patients <18 Years Old and (**B**) infants in the Kids’ Inpatient Database (2000–2009).

The use of PEG by hospital varied from 0% to 100% (Figure 1). This dramatic variability in PEG use by center was seen among both children overall and among infants in particular. In addition, variability across centers was seen among both children’s and non-children’s hospitals.

The use of the random-effect terms significantly improved the models (likelihood ratio test vs. logistic model *p* < 0.001). The estimate for the variance for the random-effect term for age <1 year was 0.46 (95%CI 0.32–0.67), demonstrating even more pronounced variability in how hospitals approach enteral access for infants.

## 4. Discussion

This study utilized a large representative national database to investigate methods for gastrostomy placement in children. SG was the more commonly utilized method, and this proportion was generally consistent over time. PEG placement was more commonly used among children with private insurance and was less commonly used among infants, children at urban hospitals, and children cared for at children’s hospitals. The use of PEG varied dramatically across centers, with some utilizing exclusively PEG and others performing exclusively SG. This variability between centers was more pronounced among infant patients.

The importance of the variability identified in this study depends upon the presence or absence of variability in outcomes between the methods of gastrostomy placement. The best method of gastrostomy placement in children is currently unknown. Several prior studies have compared the outcomes of PEG and SG, including at least three meta-analyses. The first review of 22 studies with a total of 5438 patients found a significantly increased risk of major complications with PEG compared to laparoscopic gastrostomy mostly due to a difference in visceral injury between the two techniques [3]. Another recent systematic review and meta-analysis including five retrospective studies found that while completion rates and minor complications were similar between the 550 PEG placements and 483 laparoscopic gastrostomy placements examined, significantly more bowel injuries, early tube dislodgements, and complications requiring reintervention under general anesthesia occurred after PEG placement [4]. Finally, the most recent systematic review confirmed these findings, showing that in a total of eight studies examining 1550 patients undergoing gastrostomy placement, the odds of major complications were more than three-fold higher with PEG (5.4%) compared to laparoscopic gastrostomy (1.0%) [2].

Concerns regarding the safety of PEG primarily stem from the endoscopic intra-gastric view that is utilized for PEG placement without direct visualization of adjacent organs and the inability to immediately identify injuries when they do occur, leading to delayed diagnosis and potentially increased morbidity. SG is at least theoretically safer, therefore, as it provides an intra-abdominal view to minimize injury to adjacent organs and allows for immediate identification and correction of injuries that could occur. In addition, SG allows for direct and precise placement of the gastrostomy at an optimal site on the stomach, whereas PEG placement is limited to the area on the stomach that has adequate transillumination.

Multiple studies have reported the safety of PEG placement in children, however. Brewster et al. examined 103 PEG placements performed by pediatric surgeons in children > 2 kg, identifying a total complication rate of 14%. No intra-operative complications or organ injuries were identified, although the authors acknowledged the potential limitation of their 90-day follow-up missing colonic injuries that might be identified later [9]. A prior study using the KID found that PEG and SG had similar risks of postoperative complications and mortality in relatively uncomplicated infants and neonates in whom gastrostomy was the only procedure performed [10]. PEG has the benefit of generally shorter operative time than SG. However, this difference must also be weighed against the second general anesthetic sometimes needed for the later exchange of a PEG tube for a low-profile button gastrostomy tube [4,6,11,12]. The first gastrostomy tube change after SG can typically be done in the outpatient setting without an anesthetic.

Multiple individual centers have reported a higher complication rate with PEG placements [12,13]. One review from Italy identified a higher risk of major complications after PEG (13.5%) compared to laparoscopic gastrostomy (0%) [11]. Another study from Italy prospectively evaluated complications after PEG placement in 239 children at nine centers and found that 3.3% experienced major complications that required laparotomy—six gastrocolocutaneous fistulas, one intra-peritoneal hemorrhage due to colonic injury, and one peritonitis due to tube displacement [14]. A review of 467 PEG and laparoscopic-assisted PEG placements in the Netherlands found that 12.6% of patients experienced major complications; none of these complications occurred in patients in whom laparoscopic assistance was employed [15]. The risk of complications was significantly higher in patients with ventriculoperitoneal shunts, but no association with patient age or prior upper abdominal surgery was identified. A review of PEG placements at Boston Children’s Hospital found that 10.5% of patients experienced at least one major complication [16]. Complications were again associated with the presence of a ventriculoperitoneal shunt, but smaller children (age <6 months) were found to be at *lower* risk of complications.

PEG placement in younger children, especially infants, may be particularly challenging. However, Minar et al. described successful PEG placement in 39/40 infants with a mean gestational age of 29 weeks and mean weight of 3250 g at the time of procedure. Only one major complication—an esophageal injury—was reported, although the duration of follow-up was not stated [17]. Most pediatric gastroenterologists can perform the endoscopy required for PEG placement in infants. However, the ability to perform PEG placement in infants without a trained gastroenterologist may be significantly limited. Of note, 56.1% of the PEG placements identified in this study were performed at a children’s hospital.

Safety concerns have been raised about PEG placement in younger children, highlighting the importance of patient selection. A prior study from our institution examined gastrostomy placement in infants less than one year of age and found that despite placement in a healthier cohort, PEG had more morbid and more costly complications, specifically a 3.8% risk of gastrocolic fistula, compared to laparoscopic gastrostomy [5]. In addition, Petrosyan et al. reviewed gastrostomy placements in children less than five years of age, finding that the risk of major complications with PEG (3.3%) was significantly higher than that of laparoscopic gastrostomy with or without a concomitant fundoplication (0.7%) [6]. We hypothesize that young children could be at increased risk of complications during PEG placement due to thinner tissues that are easier to transilluminate through, in particular the transverse colon or gastrocolic omentum. This difference could lead to more easily directly traversing the colon or at least the gastrocolic omentum, thereby pulling the thin colonic wall into the tract and leading to a delayed gastrocolocutaneous fistula.

This study was limited in its evaluation of outcomes. The KID captures mortality but few other outcomes, thereby limiting this study’s evaluation of potentially differential outcomes between PEG and SG. Outcomes can also vary depending on the specific technical aspects within each approach (PEG and SG), such as using fascial sutures, fluoroscopy, or transabdominal sutures [18]. Those technical details could not be ascertained given the use of ICD-9 codes in this study. In addition, given this inability to differentiate between laparoscopic and open SG, no evaluation of temporal trends or center-level variability with regard to the specific SG method was possible. Despite these limitations, this study provides a comprehensive review of the methods recently used for gastrostomy placement in children in the United States.

While gastrostomy placement is a very common procedure in children, the best method of placement has not yet been fully established. The national distribution of PEG vs. SG has remained relatively stable over time. However, the method of placement varies significantly according to patient age, insurance type, hospital location, and hospital type. In addition, centers vary dramatically with regard to their method of gastrostomy, especially within the infant population. These findings emphasize the need to further evaluate the safest method of gastrostomy placement in children, in particular among the youngest patients in whom practice currently varies the most.

## Figures and Tables

**Table 1:** Characteristics of the Study Population: Patients <18 Years of Age in the Kids’ Inpatient Database (1997–2012), Stratified by the Method of Gastrostomy Placement.

	SG (*n* = 42,980)	PEG (*n* = 24,831)	*p*-Value
PATIENT DEMOGRAPHICS			
**Mean Age** (± SD) (years)	2.3 ±4.3	4.8 ±6.0	<0.001
**Infants, *n* (%)**	25,679 (59.7)	9620 (38.7)	<0.001
0–3 months	9310 (63.3)	2742 (49.7)	
3–6 months	2509 (17.0)	1177 (21.3)	
6–9 months	1712 (11.6)	873 (15.8)	
9–12 months	1189 (8.1)	730 (13.2)	
**Female Sex**	19,554 (45.5)	11,126 (44.8)	0.4
**Race/Ethnicity, *n* (%)**			<0.001
White	18,479 (43.0)	10,846 (43.7)	
Black	5793 (13.5)	3215 (12.9)	
Hispanic	7919 (18.4)	4112 (16.6)	
Other	3832 (8.9)	2355 (9.5)	
Unknown	6957 (16.2)	4304 (17.3)	
**Insurance**			<0.001
Public	23,654 (55.0)	12,293 (49.5)	
Private	16,684 (38.8)	10,815 (43.6)	
Self-Pay	503 (1.2)	329 (1.3)	
Other/Unknown	2140 (5.0)	1394 (5.6)	
CENTER CHARACTERISTICS			
**Children’s Hospital**	24,362 (56.7)	13,931 (56.1)	<0.001
**Urban Location**	30,766 (71.6)	18,665 (75.2)	<0.001
**Teaching Hospital**	28,259 (65.7)	17,230 (69.4)	<0.001
**Bed Size**			<0.001
Small	4998 (11.6)	3321 (13.4)	
Medium	10,493 (24.4)	5736 (23.1)	
Large	25,097 (58.4)	14,730 (59.3)	
Unknown	2392 (5.6)	1044 (4.2)	
**Region**			<0.001
Northeast	5786 (13.5)	4443 (17.9)	
Midwest	9019 (21.0)	5963 (24.0)	
South	16,775 (39.0)	8120 (32.7)	
West	11,400 (26.5)	6305 (25.4)	

SG = surgical gastrostomy; PEG = percutaneous endoscopic gastrostomy; SD = standard deviation.

**Table 2:** Association of Various Patient Characteristics with the Likelihood of Undergoing Percutaneous Endoscopic Gastrostomy (PEG) versus Surgical Gastrostomy (SG) among Patients <18 Years of Age in the Kids’ Inpatient Database (2000–2009).

	OR * (95%CI)	*p*-Value
**Year** (per 3 year increment)	0.99 (0.98–1.00)	0.1
**Infant**	0.30 (0.26–0.33)	<0.001
**Female Sex**	1.02 (0.97–1.08)	0.6
**Race**		
White	Reference	
Black	1.01 (0.91–1.11)	0.9
Hispanic	1.01 (0.92–1.10)	0.9
Other	1.06 (0.95–1.18)	0.3
**Insurance**		
Public	Reference	
Private	1.17 (1.09–1.25)	<0.001
Self-Pay	1.08 (0.80–1.47)	0.6
Other/Unknown	1.14 (0.99–1.32)	0.08
**Children’s Hospital**	0.57 (0.48–0.69)	<0.001
**Urban Location**	0.38 (0.18–0.82)	0.01
**Teaching Hospital**	0.94 (0.76–1.17)	0.6
**Bed Size**		
Small	Reference	
Medium	0.92 (0.76–1.10)	0.4
Large	0.88 (0.71–1.08)	0.2
**Region**		
Northeast	Reference	
Midwest	1.21 (0.85–1.73)	0.3
South	0.88 (0.65–1.18)	0.4
West	1.12 (0.82–1.55)	0.5

* aOR: adjusted odds ratios of having PEG placement from a multiple logistic regression model adjusting for all variables shown in the table. Likelihood ratio test vs. non-hierarchical logistic model *p* < 0.001. Estimate for the variance of the random effect of infancy by hospital = 0.46 (95%CI 0.32–0.67).
